# Editorial: Unraveling renal cell carcinoma: pathogenesis, therapeutic strategies, and future perspectives

**DOI:** 10.3389/fmed.2026.1946789

**Published:** 2026-08-11

**Authors:** Wancheng Guo, Dandan Zuo, Xiulin Jiang, Hanyang Liu, Zhaokai Zhou, Ran Xu

**Affiliations:** 1 Department of Urology, The Second Xiangya Hospital of Central South University, Changsha, Hunan, China; 2 Department of Anesthesiology, Xinyang Central Hospital, Xinyang, Henan, China; 3 Department of Medicine, UF Health Cancer Center, University of Florida, Gainesville, FL, United States; 4 Department of Hepatology and Gastroenterology, Charité-Universitätsmedizin Berlin, Campus Virchow-Klinikum and Campus Charité Mitte, Berlin, Germany; 5 National Clinical Research Center for Metabolic Diseases, The Second Xiangya Hospital of Central South University, Changsha, Hunan, China

**Keywords:** clear cell renal cell carcinoma, HIF-2α inhibitor, precision medicine, renal cell carcinoma, immune microenvironment

## Introduction

Renal cell carcinoma (RCC) is a highly heterogeneous group of malignant tumors, with distinct differences among its subtypes in terms of molecular characteristics, biological behavior, and clinical response to treatment. Recently, the continuous development of anti-angiogenic agents, immune checkpoint inhibitors, and combination therapy strategies has significantly improved clinical outcomes for some patients. However, there are marked differences in treatment response among patients, and drug resistance and disease recurrence remain major factors limiting long-term benefits. Therefore, identifying key factors influencing tumor progression and treatment response is crucial for establishing more precise risk stratification and treatment strategies. The studies included in this Research Topic explore RCC from multiple perspectives, including molecular mechanisms, microenvironment regulation, precision diagnostics, and treatment optimization, further expanding our understanding of the pathogenesis, progression, and clinical management of RCC.

## Expanding the molecular landscape of renal cell carcinoma

Metabolic reprogramming, abnormal chromatin regulation, and genetic susceptibility are among the factors that contribute to tumor heterogeneity and influence disease progression and treatment response across patients (1–3). Jiang et al. developed a prognostic model based on genes related to amino acid metabolism. Further, they found that this model correlated with characteristics of the immune microenvironment, suggesting that metabolic status not only reflected adaptive changes in tumor cells themselves but also participated in regulating disease progression and the immune environment. Morales-Alvarez et al., analyzed the relationship between genetic susceptibility loci, including ITPR2, DPF3, EPAS1, and PVT1, and RCC risk and prognosis, further illustrating the varying effects of genetic variants on RCC susceptibility and progression. In addition to point mutations and susceptibility loci, chromosomal structural abnormalities also contribute to the development of RCC. Zhang et al. found that chromosomal breakpoint regions in TFE3-associated RCC frequently overlapped with non-canonical DNA structures exhibiting specific thermodynamic properties, suggesting that local genomic architecture might influence susceptibility to cancer-associated translocation events.

## Metabolism, the immune microenvironment, and treatment response

As immunotherapy has entered clinical practice for RCC, the important role of the tumor microenvironment has gradually garnered attention (4). Metabolic changes in tumor cells not only affect their own survival but can also alter treatment responses by modulating the function of immune cells (5). Hong et al. developed a prognostic assessment model combining ERO1A expression and the level of CD163^+^ tumor-associated macrophage infiltration, which improved the prediction of postoperative survival compared to traditional TNM staging. This study suggests that metabolic-related molecular characteristics and the status of immune cell infiltration may jointly influence the prognosis of RCC patients. Furthermore, Pantano et al. analyzed patients with bone-metastatic RCC treated with nivolumab and found that combination therapy with bone-targeted agents, particularly denosumab, was associated with better survival outcomes. Although this study cannot yet prove that bone-targeted therapy directly enhances the efficacy of immunotherapy, the results indicate that the bone microenvironment may play a role in modulating sensitivity to immunotherapy.

## Improving diagnosis and risk stratification through precision medicine approaches

The highly heterogeneous characteristics of RCC also complicates clinical diagnosis and treatment decision-making (6). Therefore, accurately determining the tumor's characteristics, grade, and risk of invasion is key to achieving precision therapy. Sun et al. developed a model for predicting clear cell renal cell carcinoma (ccRCC) grading that combines multi-channel, multi-phase CT radiomics with deep learning. This model can, to some extent, mitigate the information loss caused by insufficient sampling in traditional biopsies and provide supplementary information for preoperative pathological grading prediction. Additionally, for renal tumors with atypical imaging features, Gu et al. described a case of renal eosinophilic vacuolar tumor. They emphasized the importance of preoperative biopsy in avoiding unnecessary extensive surgery. In contrast, Liu et al. presented a case of papillary renal neuroendocrine tumor, demonstrating that early partial nephrectomy might achieve favorable therapeutic outcomes.

## Understanding the long-term course of the disease and personalized monitoring

Assessing the risk of postoperative recurrence of RCC is also a critical component of long-term clinical management. Some patients may still develop late-onset metastases even after curative treatment, and the sites of metastasis are not limited to common organs. Case reports by Mihailovic et al. and Wang et al. suggested that late-onset metastases might occur in patients with high-risk pathological features, even if they remain disease-free for a long time after surgery. Postoperative follow-up strategies require long-term, dynamic adjustments based on the patient's risk factors.

## Optimizing treatment strategies in the era of precision medicine

ccRCC is primarily driven by VHL deficiency and abnormalities in the HIF pathway. HIF-2α, as a key regulatory factor that becomes abnormally activated following VHL deficiency, plays a role in regulating angiogenesis, metabolic adaptation, and tumor cell survival, and is considered an important therapeutic target for ccRCC (7). Yang et al. reported that the HIF-2α inhibitor belzutifan demonstrated good efficacy and safety in patients with advanced ccRCC and suggested that the use of combination therapies warrants further exploration. Chen and Hua further noted that monotherapy with HIF-2α inhibitors may be limited by primary or acquired resistance, and that exploring combination therapy strategies is an important direction for future research. For patients with limited RCC, treatment goals have gradually shifted from maximizing tumor resection to balancing tumor control with organ function preservation. Jiang et al. and Cui et al. reported that in some complex cases, nephron-sparing surgery can still achieve good tumor control while reducing the risk of renal dysfunction. Additionally, Ye et al. found that some patients with cT1-stage RCC might experience pathological upgrading after surgery, and factors such as age, male gender, cT1b staging, and irregular tumor margins on imaging are associated with the risk of upgrading, providing a basis for preoperative risk prediction. Hu et al. conducted a population-level analysis of the disease burden and treatment outcomes of renal cell carcinoma, suggesting that disparities in the allocation of medical resources still exist across different populations and that the diagnostic and treatment needs of elderly patients require further attention.

## Conclusion

Overall, these studies included in this Research Topic collectively demonstrate a significant shift currently underway in the field of RCC research: a gradual transition from a focus on single driver gene abnormalities to a comprehensive precision medicine model that integrates genetic background, metabolic status, the immune landscape, imaging features, and treatment response. The key to future RCC research lies not only in discovering new therapeutic targets but also in elucidating how different biological characteristics collectively influence patient prognosis and treatment sensitivity. With advancements in multi-omics technologies, AI-assisted diagnosis, and novel combination therapy strategies, precision management of RCC is expected to further enhance patient outcomes. However, some current studies are still primarily based on retrospective analyses, small sample cohorts, or single-center data. Their clinical applicability requires further validation through larger-scale, multicenter, prospective studies. By integrating findings from basic research with the needs of clinical practice, it is anticipated that a more precise, dynamic, and personalized RCC management system will be established in the future.
